# Perspectives From Canadian People With Visual Impairments in Everyday Environments Outside the Home: Qualitative Insights for Assistive Technology Development

**DOI:** 10.2196/73380

**Published:** 2025-07-29

**Authors:** Prajjol Raj Puri, Andréanne Coutaller, Frédérique Gwade, Soutongnoma Safiata Kabore, Deborah Annan, Joseph Paul Nemargut

**Affiliations:** 1 Centre de Recherche Interdisciplinaire en Réadaptation du Montréal Métropolitain, Montreal, QC, Canada; 2 School of Optometry, University of Montreal, 3744 Jean-Brillant Street, Montreal, QC, H3T 1P1, Canada, 1 4385437537; 3 CISSS Montérégie-Centre, Institut Nazareth et Louis-Braille, Longueuil, QC, Canada; 4 Centre de réadaptation Lethbridge-Layton-Mackay du CIUSSS du Centre-Ouest de l’Île de Montréal, Montreal, QC, Canada

**Keywords:** visual impairment, rehabilitation, mobility, accessibility, assistive technology

## Abstract

**Background:**

Despite the abundance of assistive devices available, the accomplishment of many everyday tasks remains complex for people with visual impairments. While several studies have been conducted to identify the obstacles encountered when moving around outdoors, current knowledge is less abundant when it comes to the difficulties encountered in complex, indoor environments.

**Objective:**

This study aimed to identify the most important obstacles and facilitators encountered in everyday indoor travel environments outside the home for people with low vision and blindness.

**Methods:**

Data were collected from 20 participants with varying levels of vision from several cities across Canada in 2 web-based focus groups in both English and French. Using open-ended questions, participants shared obstacles and facilitators experienced or imagined during independent navigation in the following scenarios: coffee shop, hospital, big-box store, party with friends, and bus rides. Thematic analysis was conducted, and responses were either categorized as barriers or facilitators for each scenario. These were ranked by all participants via email according to their perceived importance in completing each scenario.

**Results:**

Across scenarios, the principal barriers to perceived success were inaccessible signage, difficulties walking around, problems finding a specific location, and unsuccessful interactions with others. The main facilitators across scenarios were helpful interactions with others, planning, accessible signage, and websites. The use of mobile apps was discussed but ranked as less important by participants. Though similar among the French and English groups, the rankings of the different facilitators and barriers were largely scenario-specific. The most barriers were mentioned in the coffee shop (n=8), followed by the department store (n=7) and bus or metro (n=7) for the English group, whereas the most barriers were in the department store (n=9), followed by the hospital or clinic (n=7) and coffee shop (n=6) for the French group.

**Conclusions:**

Though promising technologies have been developed to resolve some of the issues surrounding indoor navigation for people with visual impairments, they were not perceived as helpful as some other traditional methods of assistance, such as asking for help, by our participants. For the successful incorporation of indoor navigation technologies, it is important to understand how they integrate into the experience of people as they move in these dynamic environments. The successful use of technology is only possible if the physical environment permits and facilitates independent navigation.

## Introduction

### Perspectives of Canadian People With Visual Impairments

Globally, over 2.2 billion people have visual impairments, with projections suggesting that this number could double by 2050 [1]. More than 1,519,000 Canadian people reported having a visual disability in 2017, representing 5.4% of the population [2]. These individuals experience barriers like reading and accessing textual information [3], participating in leisure activities, shopping [3,4], and using public transport [5] that hinder their full social participation. Key tasks impacted include reading labels and signs, operating appliances, locating items on shelves, and using computers [5,6]. These barriers affect social inclusion and therefore the quality of life of people with visual impairment [2,7].

### Assistive Technology in Vision Rehabilitation

The Lancet Global Health Commission on Global Eye Health identified the provision of vision rehabilitation services and assistive technologies (ATs) as key to improving the functional abilities and quality of life of people with visual impairments [8,9]. People living with low vision or blindness often receive vision rehabilitation services from orientation and mobility specialists who instruct on the use of assistive devices that facilitate independent travel including optical aids (eg, magnifier and telescope), a long white cane for assistance with obstacle detection, and the use of a dog guide trained for obstacle avoidance while following route directions provided by the handler [10]. Low vision therapists often teach the use of assistive devices, such as screen enhancement software, magnification aids, or other visual enhancement devices to improve activities of daily living, such as reading, cooking, or watching television [11]. Vision rehabilitation therapists often provide training on screen reading software, refreshable braille displays, or other compensatory aids [12]. Once the individual has completed vision rehabilitation training, they should be able to perform many daily activities more independently or effectively, such as traveling, reading, or cooking.

Mainstream smartphones and tablets are increasingly being incorporated into vision rehabilitation training due to their built-in accessibility features (ie, screen magnification, electronic braille connectivity, and text-to-speech software) that can be activated by all users [13]. Their versatility makes them an affordable option [3], less socially stigmatizing, and generates fewer negative reactions than some specialized devices, such as electronic glasses and telescopes [14]. The accessibility features enable users to access apps that support daily living activities, including reading email, accessing websites, managing calendars, and providing GPS support during travel [15]. Recent studies have found that these modern devices are increasingly replacing the use of traditional visual aids (ie, specialized stand-alone GPS devices for blind users and optical magnification aids for those with low vision) for navigation-based tasks [14,15].

The use of these portable electronic devices enables people with visual impairments to maintain a previously unattainable level of independence [16]. Smartphone apps, like Seeing AI and Supersense, have been developed using artificial intelligence (AI)–based computer vision to provide object detection or scene description, while human assistance apps, like AIRA or Be My Eyes, may allow for remote assistance. Modern mobile GPS-based apps are often successful in facilitating outdoor navigation, allowing people with blindness or low vision to be able to plan a route, use public transportation, or identify their current location [15]. Though there are hundreds of mobile apps that could be used as AT, there is little knowledge as to their real-world benefits [17].

### AT for Social Inclusion

Despite progress in promoting inclusivity for individuals with visual impairments in design, a recent review revealed that less than 4% of studies involved them in the design and development of AT [18]. This lack of inclusion and the feeling that devices do not meet their needs often result in abandonment, a common issue in vision rehabilitation [5,18-20]. A recent study with people with visual impairments points out that 57% of everyday tasks are still performed without the support of visual aids or adaptive strategies, making it impossible to perform them without the help of another person in a third of the tasks recorded in their study [7]. Despite the wide array of indoor navigation systems tested and developed [21], indoor navigation remains a challenge for people with visual impairments [15]. To develop versatile mobile apps that respond to the needs of people with visual impairments, it is important to identify the elements of a task that do not allow them to complete it. For example, the inability to identify an item at the store would not allow them to shop independently. However, hitting an object with the white cane would not necessarily hinder their ability to shop.

One of the major advantages of modern AT is its rapid response time. Unlike vision rehabilitation specialists, who cannot provide immediate assistance, smartphones can quickly deliver vital information using AI-based computer vision, such as identifying store items. While specialists can teach users how to navigate these technologies, they cannot be present for every situation, especially in dynamic environments like restaurants or public transport. Therefore, the responsiveness of AT can significantly influence a person’s willingness and ability to participate independently in various settings. Since no single AT can address all needs, understanding the perceived barriers and facilitators among a diverse group of individuals with visual impairments is crucial.

### Research Objectives

This exploratory research investigates the common barriers and facilitators that people with visual impairments experience in everyday indoor travel environments. A Delphi-style [22] mixed methods approach was used to inform AT developers about common obstacles that are encountered among people with a variety of visual conditions and lived experiences.

Five scenarios were selected based on previously identified challenges related to using current AT in indoor settings: (1) getting a drink at your favorite local coffee shop, (2) going to the clinic for a scheduled appointment, (3) shopping at a big box store, (4) going to a party to meet friends, and (5) taking the bus.

Specifically, the research questions addressed in each of these scenarios above were as follows: (1) Which barriers hinder your ability to successfully complete the scenario? (2) What facilitates your ability to successfully complete the scenario? (3) How do mobile apps assist you in completing the scenario? (4) Which of these barriers and facilitators are the most important to achieve success in the scenario?

This is the first study, to our knowledge, that examines the perceived importance of barriers and facilitators to completing a variety of everyday indoor tasks outside the home for people with low vision and blindness.

## Methods

### Ethical Considerations

Data were collected from February through June 2023. Ethics approval consistent with the Declaration of Helsinki [23] was obtained through the Université de Montréal Clinical Research Ethics Committee (CERC 2022‐1800) on January 13, 2023. Each participant provided their informed consent to participate via an accessible Microsoft Word document sent by email. Participants were compensated US $36.50 for their time. Data collected during the study were deidentified.

### Study Design

Applying a phenomenological perspective, this research delved into the experiences of people with visual impairments in a variety of indoor, dynamic environments. This exploratory study embraces the principles of transdisciplinary development of AT [24] and user-centered design [25]. Thus, the opinions and perspectives of diverse groups of individuals with visual impairments were at the core of the study. To avoid influencing the responses of the participants, no specific technologies were mentioned throughout the study. This reduces the risk of participants being biased toward perceiving technology as the preferred method, thereby ensuring an open discussion about various means (eg, human assistance and physical accessibility measures) to resolve specific travel problems in these environments. The study methods and research findings were explicitly and comprehensively reported using the COREQ (Consolidated Criteria for Reporting Qualitative Research) checklist [26], which is presented in Checklist 1. Important details about the research team, study procedures, study setting, results, analysis, and interpretations can all be reported with the use of this checklist. To reduce question wording and personal bias in the interview, moderators AC and FG followed an interview guide that was prepared and reviewed by the principal investigator JPN prior to the focus group meetings.

### Eligibility and Recruitment

Participants were recruited between February 5 and March 26, 2023. Information was circulated by email to several organizations working with the visually impaired (eg, CNIB Foundation and Regroupement des Aveugles et Amblyopes du Montréal Métropolitain). A recruitment advertisement was also published on social media groups, such as Facebook. Finally, recruitment was carried out via a snowball effect, encouraging respondents to tell others in their circle who met the inclusion criteria about the project. The inclusion criteria consisted of choosing only participants 18 years or older of age who reside in Canada, self-identify as having a visual impairment (low vision, blindness, or deaf-blindness), are at least partially independent while traveling (make trips outside the home, independently, at least occasionally), are regular users of smartphones (at least once weekly for at least a year), can express themselves in French or English, have basic skills in using the internet and the Zoom (Zoom Video Communications) platform to participate adequately, have access to a good-quality internet connection to take part in a web-based audiovisual encounter, and declare that they had no intellectual or cognitive disabilities. Those who did not fulfill all these criteria were excluded from the study.

Individuals who expressed interest and self-identified as meeting the inclusion criteria were asked to contact the research team via the email addresses provided, whereby the research team provided the consent form in an accessible Microsoft Word format via their email correspondence provided to the researcher. A 20-minute phone or videoconference selection interview was performed to ensure the eligibility of the participants and classify them according to their age, gender, ethnicity, place of residence, travel habits, and level of vision (see Multimedia Appendix 1 for the full instrument). This information was recorded by the research team and was used to select a diverse group of participants corresponding to the categories (1-8). At least 1 participant who was classified into each category was requested to be present at each focus group. A participant may be classified into more than 1 category: (1) low vision and not legally blind, (2) low vision and legally blind, (3) low vision determined by visual acuity defect, (4) low vision determined by visual field defect, (5) low vision between 18 and 49 years of age, (6) low vision 50 years or older of age, (7) blind and between 18 and 49 years of age, and (8) blind and 50 years of age or older.

The individuals who expressed interest and were deemed eligible for selection interviews were contacted on a second occasion once they were selected by the research team based on their profile, considering the categories and encouraging the highest levels of diversity possible. The selected participants who agreed to participate in the web-based focus group were requested to respond to an accessible scheduling form to determine their availabilities, whereby the research team scheduled the web-based focus group session via Zoom.

### Data Collection and Analysis

#### Selection Interviews

For the initial data collection from the selection interview, the researchers input information regarding the age, gender, ethnic origin, place of residence, travel habits, visual diagnosis, age of diagnosis, other disabilities, smartphone use, type of mobility aid used, level of vision, type of deficit, and category (1-8).

The goal was to gather 8 to 10 people for a focus group to encourage rich, diversified exchanges, as well as the participation of everyone in a pleasant atmosphere [27]. Considering potential attrition throughout the project, 12 participants were considered for the French and English-speaking focus groups, respectively. However, due to scheduling conflicts, only 11 and 9 participants were present for the French and English focus groups, respectively. All those who participated in the focus groups completed all the remaining steps of the study.

#### Focus Group Sessions

Two focus groups were chosen to encourage diversified exchanges of ideas through Zoom meetings being conducted in French and English, respectively, to allow the participants to express their thoughts and opinions in either of the languages and to examine any potential language-related differences in the responses (ie, inaccessibility of signage or apps in 1 language). Each session, lasting a maximum of 2 hours, was moderated over Zoom by 2 members of the research team who provided the structure and rules for the session, managing time and taking notes. The moderators prepared open-ended questions and prompts to guide the conversation, but no limits were given to the type of response to be provided. All the recordings were done on the university premises, and no one else was present besides the participants and researchers during the recordings. There was no relationship established prior to the study commencement between the moderators and participants. The participants only had knowledge of the research project without any personal knowledge of either moderators AC or FG. Each of the everyday scenarios was addressed by discussing 1 subaspect at a time to help control the flow of discussion and order the data collection. For each subaspect or subtask, participants were asked to name an obstacle or facilitator or what made it harder or easier to accomplish that subtask independently. They were invited to share their opinions based on their own previous experience and expertise. If they had not experienced any of the scenarios independently in the past, they were asked to imagine themselves performing each subtask on their own. Each participant’s intervention was limited to 2 minutes per subtask to encourage as many people as possible to speak.

Once all the scenarios had been discussed, more general open-ended questions were addressed to the participants, including the strengths and shortcomings of current mobile apps, the scenarios most difficult to complete alone, and the participants’ wishes for tools to remedy the obstacles encountered.

The Zoom software was used to record the audio file of the session and transcribe it into English. The study team manually completed the French transcription. The transcriptions were validated to ensure accuracy. A third member of the research team (JPN) resolved any discrepancies after 2 team members independently identified themes in the participants’ responses. Each participant’s code, the scenario they described, and the frequency of each theme’s occurrences were recorded. The list of themes was organized according to the scenario in which it was mentioned and whether it was considered a barrier or facilitator.

#### Email Ranking

The list of themes was then emailed to each participant for an individual ranking 2 weeks after the Zoom session. The participants were only sent themes mentioned during their focus group session. Thus, each focus group had a different set of themes to choose from, but each participant in that focus group received the same set of themes. Each participant was asked to rank the themes in each scenario according to their perceived level of importance in completing the scenario independently or having a pleasant experience. They were asked to write 1 for the most important facilitator or barrier, 2 for the second most important, and so on. For each scenario, participants could also add a new idea that was not included in the proposed themes, using the “Other” line. Their responses allowed the research team to reorder the themes based on the level of perceived importance. Themes with an average value closer to 1 were determined to have a higher level of perceived importance as either a barrier or facilitator in their respective scenarios. If 2 themes received the same average value, they received the same score of relative importance.

#### Data Analysis

The audio file of the session was recorded and transcribed into English via the Zoom software. The French transcription was performed manually by the research team. Transcriptions in both English and French were validated to ensure accuracy by the principal investigator. To reduce bias, AC and FG (both female members) coded the transcripts and derived their themes independently. Themes were compared by JPN to arrive at common themes for each focus group. Discrepancies were resolved by JPN reviewing the recordings and transcripts and subsequent discussions with AC and FG. AC and FG were students in the orientation and mobility program at the Université de Montréal, while JPN (male) was their professor and research director. The code of the participant that mentioned each theme, the scenario mentioned, and the number of occurrences of each theme were logged in Microsoft Excel. The list of themes was organized according to the scenario. Each participant was asked to rank the themes in each scenario according to their perceived level of importance, with 1 being the most important. Due to the limited number of participants and high level of diversity among the participants, the authors did not perform comparative statistical tests (ie, ANOVA and *t* tests) to analyze the ranking data. Instead, the themes were analyzed according to their level of perceived importance and the information received from each participant during the selection interview. The average ranking scores and number of occurrences of themes were taken for each scenario in each language.

## Results

### Demographics

In total, 38 people were interviewed between February 24 and March 24, 2023, using the selection questionnaire. A profile of each person can be found in Multimedia Appendix 2.

The selection for each focus group was made according to the methodology described earlier to have a diverse group of participants. In total, 10 English-speaking or bilingual individuals were selected for the focus group conducted in English, and 12 French-speaking individuals for the focus group conducted in French. One individual in each group did not attend their respective focus group.

The final portrait of the 2 groups is presented in Table 1. Participants who are English-speaking have a code beginning with A, and those who are French-speaking have a code beginning with F; bilingual participants are coded AF. Participants whose codes begin with “A” or “AF” were part of the English focus group, while those with codes starting with “F” were in the French focus group. Though recorded by the research team, the visual diagnosis, travel habits, and additional handicaps are not listed in the table to deidentify the participants.

**Table 1.** Demographics of all participants in focus groups.

Code	Age (years)	Sex	Ethnicity	City, province	Age at diagnosis (years)	Mobility aids	Level of vision	Type of vision loss
A07	69	Male	White	Toronto, Ontario	Birth	None	LVB^a^	VA^b^
A10	75	Female	Asian and African	Ottawa, Ontario	68	None	LVB	VAF^c^
A18	24	Female	Afro-American	Edmonton, Alberta	12	Guide dog, white cane	LVFB^d^	VAF
AF03	62	Male	Indian	Montreal, Quebec	58	White cane	LVFB	VAF
AF08	60	Female	Middle Eastern	Toronto, Ontario	30	Guide dog, white cane	CB^e^	VAF
AF26	59	Male	White	Montreal, Quebec	Birth	White cane	CB	VAF
AF29	51	Female	White	Montreal, Quebec	Birth	Support cane, walker	LVNLB^f^	VAF
AF33	63	Male	White	Granby, Quebec	50	White cane	LVFB	VAF
AF37	30	Female	South American	Montreal, Quebec	Birth	White cane	CB	VAF
F01	50	Female	White	Montreal, Quebec	22	White cane	CB	VAF
F02	56	Female	White	Laval, Quebec	51	White cane	LP^g^	VAF
F05	50	Male	White	Mont St-Hilaire, Quebec	23	Guide dog, white cane	LVB	VAF
F20	57	Female	White	Laval, Quebec	27	White cane	LVB	VAF
F23	58	Female	White	Laval, Quebec	57	White cane	LVB	VF^h^
F25	66	Male	White	Quebec, Quebec	55	White cane	LP	VAF
F27	32	Female	African	Longueuil, Quebec	8	White cane	CB	VAF
F28	61	Male	White	Granby, Quebec	20	Guide dog, white cane	LP	VAF
F30	56	Female	White	Montreal, Quebec	Birth	White cane, support cane, telescope	LVNLB	VA
F35	58	Female	White	St-Hubert, Quebec	32	White cane	LVFB	VAF
F36	56	Female	White	Granby, Quebec	17	None	LVB	VA

a LVB: low vision and legally blind.

b VA: visual acuity loss.

c VAF: visual acuity and field loss.

d LVFB: low vision functionally blind.

e CB: completely blind.

f LVNLB: low vision not legally blind.

g LP: light perception.

h VF: visual field loss.

### Coffee Shop

The participants were asked to imagine getting a drink at their favorite coffee shop, and this scenario consisted of multiple steps (Multimedia Appendix 3). In each of the steps of this scenario, the participants in both focus groups were asked to indicate any obstacles and facilitators. They were then asked to rank these themes in an email. This process was repeated in each scenario. A heat map summary including the full list of themes along with the ranking of each across all scenarios is shown in Multimedia Appendix 4.

The themes extracted for the facilitators and barriers were reviewed and ranked by participants. The complete list of ranking of themes for this and each scenario are presented in Multimedia Appendix 5. The top 3 rankings of the barriers and facilitators in each group for the coffee shop scenario, and their averages, are indicated in Table 2.

Participant AF37 mentioned that human assistance is an important factor, stating that “If I go to a known place, I already know kind of the prices on what they sell, and so I just ask them, what do you have today?” More quotes on each theme used by participants are mentioned in Multimedia Appendix 6.

Participant AF08 mentioned that the precise location is difficult to find, stating

Locating where the terminal is very difficult. It’s never in a standard place that is close to the customer. Sometimes you must reach and try to find where it is.

**Table 2.** Top 3 rankings of facilitators and barriers in order of importance to visit a coffee shop.

Rank	English group	Average (SD)	French group	Average (SD)
Facilitators—coffee shop				
1	Human assistance	2.6 (2.0)	Human assistance	1.9 (0.9)
2	Preparedness	3.4 (1.7)	Accessible payment	2.4 (1.1)
3	Accessible physical space	4.3 (1.6)	Sensory cues	2.6 (1.0)
Barriers—coffee shop				
1	Difficulty finding an exact location	2.7 (1.8)	Lighting	2.0 (1.5)
2	Unsuccessful interactions with staff	3.1 (1.2)	Inaccessible signage	2.5 (1.0)
3	Inaccessible signage	3.8 (2.5)	Difficulty walking around	2.9 (1.1)

### Hospital

The participants were then asked to imagine going to a hospital or a clinic for their regular checkup (Multimedia Appendix 3). The top 3 rankings, as determined by participants’ averaged ranking scores, of the barriers and facilitators in each group are indicated in Table 3.

Participant AF28 mentioned that it was convenient to use human assistance, as he quoted, “Ask the security guard to go to the right floor. Then ask the receptionist and tell him or her.”

Participant A07 pointed out the problem of finding a precise location by mentioning:

You’ve got to find the right floor. Just find the elevator. There are all kinds of challenges with that if you want to go a big place like, say, St. Michael’s Hospital, and you have to go up to floor 8 or 10, which is very difficult to pinpoint.

**Table 3.** Top 3 rankings of facilitators and barriers in order of importance to visit a hospital or clinic.

Rank	English group	Average (SD)	French group	Average (SD)
Facilitators—clinic				
1	Human assistance	1.4 (0.5)	Human assistance	2.1 (1.0)
2	Mobility aid	2.0 (0)	Preparedness	2.1 (1.2)
3	Preparedness	2.2 (1.0)	Accessible signage	2.7 (0.8)
Barriers—clinic				
1	Difficulty finding an exact location	1.9 (0.9)	Inaccessible signage	2.4 (1.4)
2	Unsuccessful interactions	2.3 (1.0)	Unsuccessful interactions	2.9 (1.4)
3	Difficulties walking around	2.6 (1.1)	Difficulty walking around	3.1 (1.4)

### Big Box Store

The participants were then asked to imagine shopping in a big box department store (Multimedia Appendix 3). The top 3 rankings of the barriers and facilitators in each group, and their averages, are indicated in Table 4.

Participant A10 mentioned the importance of human assistance by saying, “If you are making a comparison between items and prices. I find that having human intervention helps.”

Participant AF33 mentioned the problems locating a precise item, quoting that “One thing that makes it difficult for me is, is it’s either the top row or completely the bottom row.”

**Table 4.** Top 3 rankings of facilitators and barriers in order of importance to shop at a department store.

Rank	English group	Average (SD)	French group	Average (SD)
Facilitators—shopping				
1	Human assistance	2.6 (1.9)	Website accessibility	1.9 (0.9)
2	Preparedness	3.9 (2.6)	Preparedness	2.5 (1.7)
3	Website accessibility	4.1 (2.0)	Human assistance	3.2 (1.9)
Barriers—shopping				
1	Difficulties walking around	2.8 (1.6)	Employee assistance request	1.0 (0)
2	Difficulty finding an exact location	3.1 (1.3)	Inaccessible signage	2.4 (1.5)
3	Problems locating a precise item	3.6 (1.6)	Difficulties walking around	2.5 (1.6)

### Party

The participants were then asked to imagine going to a party with friends (Multimedia Appendix 3). The top 3 rankings of the barriers and facilitators in each group, and their averages, are indicated in Table 5.

Participant AF37 mentioned how help from friends can be an immense help to join a party by quoting:

I usually call when I am arriving, and how my friend, or whoever can you please meet me downstairs? That would be helpful because I usually will not know where the house is.

Participant AF37 also mentioned how unsuccessful interaction with friends creates more difficulty by quoting:

For me the challenge is that in some kind, of course, people are moving around all the time, you know, going from group to group to group to group, and it’s very over overwhelming to kind of follow that it’s someone I know, and even work with there’s people that I don’t know, because I cannot pinpoint, or it’s very hard for me to kind of just join a random group.

**Table 5.** Top 3 rankings of facilitators and barriers in order of importance while going to a party with friends.

Rank	English group	Average (SD)	French group	Average (SD)
Facilitators—party				
1	Preparedness	2.0 (1.2)	Human assistance	1.2 (0.4)
2	Accessible physical space	2.4 (0.7)	Accessible physical space	2.7 (1.1)
3	Human assistance	2.6 (1.3)	Alternatives available	3.1 (1.2)
Barriers—party				
1	Difficulties walking around	2.6 (1.42)	Fear of spilling food or drink on others	2.0 (0.5)
2	Unsuccessful interactions	3.0 (1.7)	Noisy environment	2.5 (1.2)
3	High ambient noise	3.4 (2.2)	Difficulties walking around	2.5 (1.2)

### Bus Travel

The participants were then asked to imagine going on public transportation such as a bus or metro station (Multimedia Appendix 3). The top 3 rankings of the barriers and facilitators in each group, and their averages, are indicated in Table 6.

Participant A10 mentioned what preparations could be made to make traveling on the bus more convenient, quoting:

There’s a lot of research to be done ahead of time. You must know the stop numbers. You must know the intersections where those bus stops are. These are things you must know before getting there.

Participant AF33 mentioned that the inaccessible signage was one of the major barriers, quoting that:

One thing is that the sign is way too high. It is sometimes 8 to 12, and even 14 feet high. I cannot reach it by phone to be able to see if I am at the right spot.

**Table 6.** Rankings of facilitators and barriers in order of importance while going on public transportation.

Rank	English group	Average (SD)	French group	Average (SD)
Facilitators—public transit				
1	Accessibility of signage	2.8 (1.8)	Accessible signage	1.9 (1.1)
2	Preparedness	3.00 (2.2)	Accessible physical space	2.4 (1.1)
3	Accessible physical space	3.0 (1.3)	Human assistance	2.8 (1.5)
Barriers—public transit				
1	Inaccessible signage	2.1 (1.9)	Inaccessible signage	1.4 (0.9)
2	Unsuccessful interactions with staff or patrons	3.7 (2.2)	Difficulty finding an exact location	2.4 (0.8)
3	Difficulties walking around	3.9 (1.5)	Unexpected events	2.5 (1.3)

### Scenario Comparison

Participants were asked which scenario was the most difficult to navigate on their own and were asked to explain their reasoning. The most barriers for the English group were indicated in the coffee shop (n=8) followed by department store (n=7) and bus or metro (n=7). For the French group, the most barriers were in the department store (n=9), followed by the hospital or clinic (n=7) and coffee shop (n=6). The fewest facilitators were mentioned in the hospital or clinic (n=5) for the English group, while it was the coffee shop (n=4) for the French group. For the French group, the department store had one of the largest barriers (n=9) and the largest number of facilitators (n=8). Meanwhile, the coffee shop scenario had both the most barriers (n=8) and facilitators (n=8) for the English group. Elaborated data are presented in graphs in Tables7  8.

Among the facilitators identified, human assistance was consistently regarded as a major factor being present in all scenarios. Accessibility of physical space was also a significant facilitator observed in most scenarios (4 times in English and 5 times in French). Preparation was noted as a facilitator in 5 English and 2 French scenarios.

Regarding major barriers, nonaccessible signage and difficulty walking around were reported in almost all scenarios (5 times in English and 4 times in French). Unsuccessful interactions with others were identified in 4 English and 4 French scenarios. Difficulty locating precise locations also emerged as a barrier in 4 English and 4 French scenarios. Details for each scenario are tabulated in Multimedia Appendix 5.

When comparing the French and English groups, they were similar. While the groups perceived an equal number of barriers, the French group experienced fewer facilitators. The English group considered smartphone apps as one of the facilitators in every 5 scenarios, while the French group considered it as a facilitator in only 2 scenarios. Similarly, the English group seemed to be prepared and found preparedness as a major facilitator, while the French group pointed out preparedness as a facilitator in only 2 scenarios. Even though both groups had an equal number of barriers, nonaccessible signage, as well as difficulty walking around, were present in all 5 scenarios for the English group, while it was present in 4 scenarios for the French group.

**Table 7.** The number of barriers and facilitators experienced by people with visual impairments in each scenario among the English group.

English group	Barriers experienced, n	Facilitators experienced, n
Coffee shop	8	8
Hospital	4	5
Shopping	7	8
Party with friends	6	7
Public transportation	7	7

**Table 8.** The number of barriers and facilitators experienced by people with visual impairments in each scenario among the French group.

French group	Barriers experienced, n	Facilitators experienced, n
Coffee shop	6	4
Hospital	7	5
Shopping	9	8
Party with friends	5	5
Public transportation	5	6

### Use of Smartphone

To understand how smartphones can bring independence to people with visual impairments, we asked five questions, which were (1) What are the strengths and weaknesses of the currently available apps that you use? (2) What is missing from the currently available apps for the scenarios we described? (3) What would the ideal mobile app contain? (4) Among the above-given scenarios, in which situation would you prefer an app and in which one would you prefer human assistance? (5) Which of the above scenarios was the most difficult to navigate on your own?

For the first question, the participants provided examples of a few smartphone apps to share their strengths, such as being able to have voice commands and being user-friendly. Participants went on to discuss apps, such as Seeing AI, Google Maps, Soundscape, and BlindSquare, which they found especially useful and user-friendly. However, each app had its weaknesses. It was difficult to switch from one tab to another in Seeing AI. Google Maps is unable to work in indoor environments, and accuracy depends somewhat on the weather conditions. Soundscape, though, was very promising and user-friendly but was discontinued and required training. Similarly, the BlindSquare app also required training, which made the work tiring.

For the second question, participants explained that most of the apps do not have accessibility options like a back button. Furthermore, there was an issue with the GPS not being accurate, and a few participants mentioned the difficulty of navigating indoors. Additionally, all participants agreed that each app is incomplete and that to do each different task, they would have to switch between the different apps, which was troublesome. Further, they found it difficult to hold their phones to scan most of the time and advised creating a method with which they could be hands-free, which might not be as expensive as smart glasses.

For the third question, participants agreed that the ideal content would be to have all the features in 1 app so that they would not have to switch between the apps. Other ideal features included voice commands and hands-free operation. They also wanted the software to be sustainable and not drain excessive batteries from their phone.

For the fourth and fifth questions, there was a split in opinion among participants, while most of them agreed that any of them would love to have an app on their phone that would allow them to successfully navigate any of the given scenarios, a few participants expressed doubts about the feasibility of an app enabling independent travel in certain scenarios due to the complexity of tasks required in those environments.

More participant quotes can be found in Multimedia Appendix 6.

## Discussion

### Principal Findings

People with visual impairments encounter numerous issues in daily life that reduce their participation in social and leisure activities. The goal of our study was to better understand the perceived barriers and facilitators for everyday travel for 5 of these activities outside the home for people living with both low vision and blindness. This study was particularly focused on elucidating the context of the problems associated with everyday indoor travel environments to inform technology developers about priorities from those with a variety of lived experiences. This study takes a user-centered approach to enumerate perceptions and opinions even for individuals who may not be able to complete these tasks independently. Here, we discuss some of the major points that were prioritized by individuals in our focus groups and examine how technology developers may attempt to reduce the barriers while facilitating the use of mobile apps and other technologies to respond to the needs of people with visual impairments in the environments studied.

### Barriers

Nonaccessible signage and difficulty walking around emerged as major concerns and were addressed in almost all scenarios. Particularly, inaccessible signage contributes significantly to the challenge of accessing written information for people with visual impairments, as highlighted in studies by Brown et al [23] and da Silva et al [3]. Several attempts have been made to improve the readability of signage for this population. For instance, Lan et al [28] developed lightweight smart glass equipped with a tiny computer to assist users in reading signage. While this technology proved helpful in reading street signs, it was not tested in people with visual impairments, and its functionality was limited to this single purpose. This might explain why the product has never reached the market yet. Similarly, Crandall et al [29] created an infrared audible signage system. However, it failed to gain popularity due to its complex sensor installation requirements and lack of usability. Even after training, the voice output was difficult for people with visual impairments to understand. To develop user-friendly technology, it is essential to involve end users from the prototype stage and conduct regular testing and refinements until the product meets their needs and ensures their comfort and satisfaction.

The theme of difficulties walking around, highlighted by the participants in our study, echoes the findings of Lamoureux et al [30]. For people with visual impairments, this remains a significant obstacle. One reason this challenge persists is that many systems are designed to either assist with outdoor navigation or indoor navigation, but rarely both. Transitioning between indoor and outdoor spaces is particularly difficult, possibly due to the challenge of recognizing entry and exit doors, sudden changes in lighting, the sensory overload caused by varying sounds and environmental conditions, and the absence of consistent orientation cues between these settings. The difficulty in finding a precise location and unsuccessful interactions with employees, volunteers, and other people present were identified as major obstacles in the proposed scenarios. Recognizing precise locations indoors is particularly challenging, as indoor environments hinder many sensory cues, and the widely used GPS does not function effectively indoors. To address these challenges, various alternative solutions have been explored, such as a guide beacon [31], Wi-Fi fingerprinting [32], and live streaming [33]. Each of these methods comes with its own advantages and limitations. Guide beacons, which are low-cost Bluetooth devices, must be strategically installed throughout the indoor spaces. Wi-Fi fingerprinting relies on consistent Wi-Fi signals and detailed floor mapping to operate effectively. Live streaming, on the other hand, requires a person to view the stream and provide location guidance, which is often impractical, inefficient, and expensive. Further research in these areas might provide us with better technology for overcoming these obstacles.

While human assistance was often a facilitator, unsuccessful interactions with employees emerged as recurring barriers in different scenarios. To improve social interaction, a Social-Aware Assistant was developed that could provide people with visual impairments cues to enhance their face-to-face interaction using a smart glass and haptic belt [34]. To achieve a level of independence for people with visual impairments, ATs and human assistance are inseparable [35]. Based on this, several attempts have been made such as FaceTime [36], AIRA [37], Be My Eyes [38], and Guide Call [39] to use human assistance in assistive devices. Even though these attempts have been made, they have not been widely popular during these scenarios. The major reason for this is being expensive if human agents are used and the unavailability of assistance when volunteers are used.

### Facilitators

To overcome the obstacles encountered, the participants identified several strategies and tools that they use and the elements that assist them in accomplishing these everyday tasks. The findings indicate that the assistance of staff, volunteers, clients, or other bystanders is the most frequently used strategy, as identified in all 5 scenarios in each of the focus groups. This reliance on human assistance aligns with the results of the 2017 Canadian Survey on Disability [40], which reports that 58% of people with disabilities, including those with visual impairments, receive help with daily activities such as shopping or attending appointments. However, this dependence on others can be unsettling for people with visual impairments, as it limits their sense of independence. Therefore, technology could offer more empowering alternatives, allowing people with visual impairment to decide when to seek assistance. Some participants even expressed a preference for AI-based assistance over human help, as it made them feel more comfortable and in control.

Using smartphones with different functions was also one of the major facilitators. There is a huge improvement in technology, specifically in the smartphone, which has enabled several functions enabling people with visual impairments to perform most of their tasks conveniently. There are several apps designed for people with visual impairments with specific functions. Functions like zoom-in, improved camera quality, and voice command are the features that people with visual impairments mostly use. Mobile apps that were launched recently, such as Blind-Aid [41], HandyApps [42], AKSHI [43], and ARIANNA [44], which included multiple features for navigation for people with visual impairments, could be useful but were not mentioned by those in our study. Furthermore, there are other apps designed for specific tasks such as Farmaceutic App [45] for drug information, TransmiGuia app [46] for public transportation, and money-recognizing apps [47] could be useful as well. It was also interesting to find that smartphone apps were mostly found useful by the English group as opposed to the French group, which may be due to the language barriers provided in most of the apps. Mobile app creators should be conscious of the accessibility features in each of the available languages when creating and updating an app.

Preparedness emerged as an important facilitating strategy among study participants, alongside having an accessible environment and clear signage. Many participants relied on Google Maps and a mental image of the places they had previously visited. However, when traveling to unfamiliar locations, especially within complex indoor environments, there remains a lack of sufficient technology to assist people with visual impairments. Indoor maps are particularly challenging to create and maintain due to the complexity and frequent layout changes, making it difficult for existing technologies to offer reliable indoor navigation maps. Recently, SITUM [48] has been working to address this issue by developing comprehensive indoor mapping solutions through extensive global research. Furthermore, preparedness was found to be an important facilitator in the English group being present in all 5 scenarios but was only present in 2 scenarios in the French group. This might be linked with the use of smartphones, as preparation is mostly associated with smartphone use.

Signage is a key facilitator, benefiting both individuals with visual impairments and those with normal vision. To make signage more accessible, Rousek and Hallbeck [49] concluded that simple, high-contrast pictograms, depicting human figures, were most easily recognized by participants wearing glasses that simulated different visual conditions. They also recommend standardizing signage within hospitals to facilitate the cognitive processes related to understanding signs. Their findings can certainly be applied to written signage in complex environments.

While several facilitators are used to navigate the obstacles experienced in various environments, some are implemented to avoid environments where these obstacles are particularly overwhelming. One such solution, adopted by many participants, is web-based shopping, which enables them to bypass department stores and grocery stores altogether. The ability to select items on the web and have them delivered at home not only eliminates the need for external assistance but also reduces the fatigue associated with in-store shopping. The significant benefit of web-based shopping for people with visual impairments is also highlighted by Kostyra et al [50], who studied the barriers and facilitators reported by people with visual impairments, including when visiting grocery stores. Indeed, they already pointed out a few years ago that the development of web-based shopping was a promising avenue for improving the satisfaction and independence of blind people, which is confirmed by our results. However, website accessibility remains an issue associated with web-based shopping for people with visual impairments.

### Recommendations for Developers

The findings of this study offer direct implications for the design and refinement of ATs for people with visual impairments. Below, we outline several recommendations drawn from participant insights.

#### Human Interaction

In noisy, dynamic spaces like parties or coffee shops, participants described challenges in initiating and maintaining social interactions. AT could integrate features like facial expression recognition, conversational cueing, or proximity alerts to help users locate and engage with others more confidently.

#### Improve Indoor Navigation Capabilities

Participants identified difficulties in locating precise destinations indoors as a major barrier. Since GPS is often unreliable indoors, alternative technologies like Wi-Fi fingerprinting, Bluetooth beacons, or AI-powered computer vision should be explored to enhance spatial awareness and positioning accuracy.

#### Design for Multifunctionality and Hands-Free Use

Several participants expressed their frustration with switching between single-purpose apps. Developers should aim to integrate multiple features like object recognition, Optical Character Recognition, GPS, and communication support within a single platform. Voice-controlled or wearable solutions may further facilitate hands-free use and improve user experience.

#### Language and Accessibility Gaps

Participants from the French-speaking group noted that some apps lacked full functionality in French. Inclusive AT design must ensure multilingual support, compatibility with screen readers, and braille display integration to accommodate users across linguistic and accessibility needs.

### Limitations

The data were only collected from people with visual impairments living in Canada, and most of our participants were from Quebec. Further research could collect data from all the provinces of Canada or other parts of the world to provide richer data. Since the data were collected from web-based interviews, it was only accessible to the individuals who had internet access and could use videoconferencing technology independently. In addition, though the sample size was diverse, it does not necessarily represent the views and opinions of many other Canadian people with visual impairments. Larger datasets around the world could help the AT industry to have better ideas of the requirements of people with visual impairments in their lived environments.

### Conclusions

The results provide a better understanding of the realities of people with visual impairments in indoor, dynamic environments. Our results highlight that mobile technologies, though while facilitating, are not as critical to completing multiple everyday tasks outside the home. Human assistance, accessibility of physical space, and preparedness were found to be major facilitators but are considered barriers when absent or inaccessible. Smartphone apps should be considered within the larger context of completing daily tasks, as they may provide some assistance but will not be the universal solution. Additionally, language barriers must be considered, as many apps often do not have the full range of functionality in both languages, thus leading to a disadvantaged population of nonnative English speakers who may have encountered additional barriers, such as regarding preparedness. Further research should examine how mobile technologies perform when individuals are interacting with everyday environments outside the home in the context of other human factors (ie, communication) and accessibility of physical space (ie, available signage).

## Supplementary material

10.2196/73380 Multimedia Appendix 1 Questionnaire for selection of participants for focus group.

10.2196/73380 Multimedia Appendix 2 Profiles of 38 people interviewed, using the selection questionnaire.

10.2196/73380 Multimedia Appendix 3 Scenarios and subtasks.

10.2196/73380 Multimedia Appendix 4 Summary heat map of the facilitators and barriers (rows) in the French and English focus groups for each scenario (columns), according to their ranking order, as determined by the average responses from participants.

10.2196/73380 Multimedia Appendix 5 Complete rankings of the facilitators and barriers in order of importance to visit different scenarios for the English and the French focus groups.

10.2196/73380 Multimedia Appendix 6 Example quotes from participants for each theme.

10.2196/73380 Checklist 1 COREQ (Consolidated Criteria for Reporting Qualitative Research) checklist.

## Abbreviations

AI: artificial intelligence
AT: assistive technology
COREQ: Consolidated Criteria for Reporting Qualitative Research
