# Supplementary material for: Perspectives From Canadian People With Visual Impairments in Everyday Environments Outside the Home: Qualitative Insights for Assistive Technology Development
Source: JMIR Rehabil Assist Technol. 2025 Jul 29;12:e73380. doi: 10.2196/73380 (PMC12306908; doi:10.2196/73380)
Supplement: Multimedia Appendix 1 [file rehab-v12-e73380-s001.docx]

**To participate in this research project, the participant must:**

- have read and accepted the consent form

- be at least 18 years of age

- self-identify as visually impaired (blind, low vision or deaf-blind)

- self-identify as being at least semi-independent when travelling (travelling independently at least occasionally)

- be able to participate in a virtual discussion group

- self-identify as having moderate to high experience and frequent use of a smartphone

- have no self-reported cognitive or intellectual impairment

- be able to communicate in French and/or English

**Participant Selection and Participation Questionnaire**

(In the form of an interview with a vision rehabilitation professional)

1. What is your current age?

2. What gender do you identify with?

3. What is your ethnic background?

4. In which Canadian city and province do you live?

5. Where do you travel most of the time (e.g. urban, semi-urban/suburban, rural)?

6. What is your visual diagnosis (if you know)?

7. At what age did your vision impairment begin, if known?

8. In your own words, describe your vision level.

To guide the response to question 8, the following follow-up questions (to categorize the participant)

8a. When traveling, do you have difficulty identifying and avoiding obstacles?

8b. Do you ever use optical aids (e.g., glasses, magnifying glass, television)?

8c. Do you receive the Federal Disability Tax Credit (DTC)? (legal blindness or other disability)

8d. Do you perceive light?

***For the professional***

The participant may be categorized as follows:

A- Low vision not legally blind

B- Low vision and legally blind

C- Functionally blind with low vision

D- Blindness with light perception

E- Complete blindness

Primarily, the participant's vision shows impairment of:

A- visual acuity

B- visual fields

C- visual acuity and visual fields

D- neurological condition

________________________________________________

9. Do you have a disability other than visual (e.g. hearing, motor, etc.)?

10. What mobility aid (other than smartphone) do you use (e.g. white cane, guide dog, optical aid) ?

11. How often do you make independent trips outside your home? (e.g. daily, a few times a month)

**Participant contact information**

First name:

Last name:

Telephone number:

Email address:

**_______________________________________________________________**

**Professional’s analysis**

**The participant meets all of the inclusion criteria listed above**

__Yes

__No

**Participant Profile (Check all that apply)**

__Low vision and not legally blind

__Low vision and legally blind

__Low vision determined by visual acuity defect

­__Low vision determined by visual field defect

__Low vision between 18 and 49 years of age

__Low vision 50 years of age or older

__Blind and between 18 and 49 years of age

__Blind and 50 years of age or older
