# Supplementary material for: Perspectives From Canadian People With Visual Impairments in Everyday Environments Outside the Home: Qualitative Insights for Assistive Technology Development
Source: JMIR Rehabil Assist Technol. 2025 Jul 29;12:e73380. doi: 10.2196/73380 (PMC12306908; doi:10.2196/73380)
Supplement: Multimedia Appendix 2 [file rehab-v12-e73380-s002.docx]

Profile of thirty-eight people interviewed between February 24 and March 24, 2023, using the selection questionnaire are tabulated below

| Code | Age | Gender | Ethnicity | City and province | Age of onset | Smartphone | Mobility aids used | Vision Level | Visual impairment | Participant Categories |
| --- | --- | --- | --- | --- | --- | --- | --- | --- | --- | --- |
| A04 | 48 | F | Middle Eastern | Toronto, ON | Birth | iPhone | Guide dog, Cane (for some occasions) | BVF | ACV | B, C, D, E |
| A07 | 69 | H | Caucasian | Toronto, ON | Birth | iPhone | No | BVL | AC | B, D, F |
| A09 | 39 | F | Asian | Toronto, ON | Birth | iPhone | White cane | BVF | ACV | B, C, D, E |
| A10 | 75 | F | Asian and African | Ottawa, ON | 68 | iPhone | No | BVL | ACV | B, C, D, F |
| A12 | 70 | H | Caucasian | Beaconsfield, Qc | Birth | iPhone | White CaneInvason Smart Glasses | PL | ACV | B, C, D, FouH |
| A15 | 25 | F | Asian | Toronto, ON | Birth | iPhone | Guide dog, cane | CC | ACV | G |
| A16 | 46 | H | Asian | Toronto, ON | Birth | iPhone | White cane | PL | ACV | G |
| A17 | 54 | F | South American | Toronto, ON | 5 | iPhone | Guide dog | CC | ACV | H |
| A18 | 24 | F | African American | Edmonton, Alberta | 12 | IPhone | Guide dog, cane | BVF | ACV | B, C, D, E |
| AF03 | 62 | H | Indian | Montreal, QC | 58 | iPhone | White cane | BVF | ACV | B, C. D. F |
| AF08 | 60 | F | Middle Eastern | Toronto, ON | 30 | iPhone | Guide dog, cane | CC | ACV | H |
| AF11 | 48 | F | Causaienne | Laval, Qc | Birth  Retinal detachment at age 40 | iPhone | White cane | BVL | ACV | B, C, D, E |
| AF26 | 59 | H | Causaienne | Montreal, QC | Birth | iPhone | White cane | CC | ACV | H |
| AF29 | 51 | F | Caucasian | Montreal, QC | Birth | 2 | Support cane, walker | BVNL | ACV | Has |
| AF31 | 22 | F | Caucasian | Mascouche, Qc | Birth | IPhone | White cane, guide dog | CC | ACV | G |
| AF33 | 63 | H | Caucasian | Granby, Qc | 50 | IPhone | White cane for unknown places | BVF | ACV | B, C, D, F |
| AF37 | 30 | F | South American | Montreal, QC | Birth | IPhone | White cane | CC | ACV | G |
| F01 | 50 | F | Caucasian | Montreal, QC | 22 | iPhone | White cane | CC | ACV | H |
| F02 | 56 | F | Causasian | Laval, Qc | 51 | iPhone | White cane | PL | ACV | H |
| F05 | 50 | H | Causasian | Mont St-Hilaire, Qc | 23 | iPhone | Guide dog, white cane | BVL | ACV | B, C, D, F |
| F06 | 46 | F | Caucasian | Trois-Rivières, Qc | 38 | iPhone | White cane | BVF | ACV | B, C, D, E |
| F13 | 45 | F | Caucasian | Ottawa, ON | Birth | iPhone | White Cane, Echolocation | CC | ACV | G |
| F14 | 71 | H | Caucasian | Montreal, QC | 60 | Android, Samsung | White cane | BVL | ACV | B, C, D, F |
| F18 | 56 | F | Caucasian | Montreal, QC | 25 | iPhone | White cane | PL | ACV | H |
| F19 | 25 | H | South American | Montreal, QC | 7 | Android, Samsung | White cane | BVL | ACV | B, C, D, E |
| F20 | 57 | F | Caucasian | Laval, Qc | 27 | iPhone | White cane | BVL | ACV | B, C, D, F |
| F21 | 39 | F | Caucasian | Montreal, QC | 30 | iPhone | White cane, flashlight | BVL | CV | B, D, E |
| F22 | 56 | H | Causasian | Longueuil, Qc | Birth | Android, TCL (Best Sound) | White cane | BVL | ACV | B, C, D, F |
| F23 | 58 | F | Caucasian | Laval, Qc | 57 years old | iPhone | White cane | BVL | CV | B, D, F |
| F24 | 34 | H | Caucasian | Montreal, QC | Birth | 2 | White Cane, Victor Reader Trek | CC | ACV | G |
| F25 | 66 | H | Caucasian | Quebec City, QC | 55 | IPhone | White cane | PL | ACV | H |
| F27 | 32 | F | African | Longueuil, Qc | 8 | IPhone | White cane | CC | ACV | G |
| F28 | 61 | H | Caucasian | Granby, Qc | 20 | IPhone | Guide dog, white cane | PL | ACV | H |
| F30 | 56 | F | Caucasian | Montreal, QC | Birth | IPhone | Detection rod, support rod, telescope | BVNL | AV | Has |
| F32 | 66 | H | Caucasian | Montreal, QC | 15 | IPhone | Telescope, solar filters | BVL | AV | B, C, F |
| F34 | 38 | H | Caucasian | Brossard, Qc | 20 | IPhone | White cane, sun filters | BVL | CV | B, D, E |
| F35 | 58 | F | Caucasian | St-Hubert, Qc | 32 | IPhone | White cane | BVF | ACV | B, C, D, F |
| F36 | 56 | F | Caucasian | Granby, Qc | 17 | Android | No | BVL | AV | B, C, F |
