# Supplementary material for: Perspectives From Canadian People With Visual Impairments in Everyday Environments Outside the Home: Qualitative Insights for Assistive Technology Development
Source: JMIR Rehabil Assist Technol. 2025 Jul 29;12:e73380. doi: 10.2196/73380 (PMC12306908; doi:10.2196/73380)
Supplement: Multimedia Appendix 3 [file rehab-v12-e73380-s003.docx]

| 1. Pick up a beverage at a **coffee shop**  - Identify the business  - Enter the business  - Queue up  - Read the menu and check prices  - Order the drink and snack of your choice  - Make the payment  - Get sugar and/or milk at the station provided for this purpose (if applicable)  - Identify an available table and sit down  - Drink your beverage  - Arrange the disposable cup or cup properly  - Leave the business |
| --- |
| 2. Go to a **clinic or hospital** appointment  - Entering the clinic  - Go to the reception  - Head to the waiting room  - Find an empty seat and sit down  - When your name is called, go to the appropriate room  - Enter the appropriate room  - Interact with the professional for the reasons of the appointment  - Leave the room and go to the reception area.  - Make the payment  - Leave the clinic |
| 3. Shop at a **big-box store**  - Enter the store  - Take a shopping cart, if needed  - Understand the layout of the store  - Move through aisles towards the departments of interest  - Identify products and make the right choice  - View item prices and discounts  - For the purchase of clothes, go through a fitting room to determine if they are suitable  - Head to the checkouts  - Complete the transaction with the cashier or through a self-service checkout by following the necessary steps |
| 4. Celebrate at a **party**  - Go to the address where the party is taking place  - Familiarize yourself with the place  - Identify known people/friends on site  - Interact with those present  - Identify available food and beverages  - Help yourself to food and drink  - Find your drink among the others  - Socialize with new people  - Understand and participate in conversations despite loud music  - Move and dance at an appropriate distance from others  - Have fun |
| 5. Use of the **bus**  - Buy tickets for your "transport" card  - Getting to the bus stop of your choice  - When the bus arrives, make sure it is the one you expect  - Getting on the bus  - Present your card in the right place to pay for the passage  - Move forward on the bus and locate an available seat  -Sit  - Pay attention to the announcement of stops  - When the desired stop is announced, press a button to signal the driver to stop  - Head to the bus exit  - At the stop, open the exit door and get off the bus |
