# Supplementary material for: Perspectives From Canadian People With Visual Impairments in Everyday Environments Outside the Home: Qualitative Insights for Assistive Technology Development
Source: JMIR Rehabil Assist Technol. 2025 Jul 29;12:e73380. doi: 10.2196/73380 (PMC12306908; doi:10.2196/73380)
Supplement: Multimedia Appendix 4 [file rehab-v12-e73380-s004.pdf]

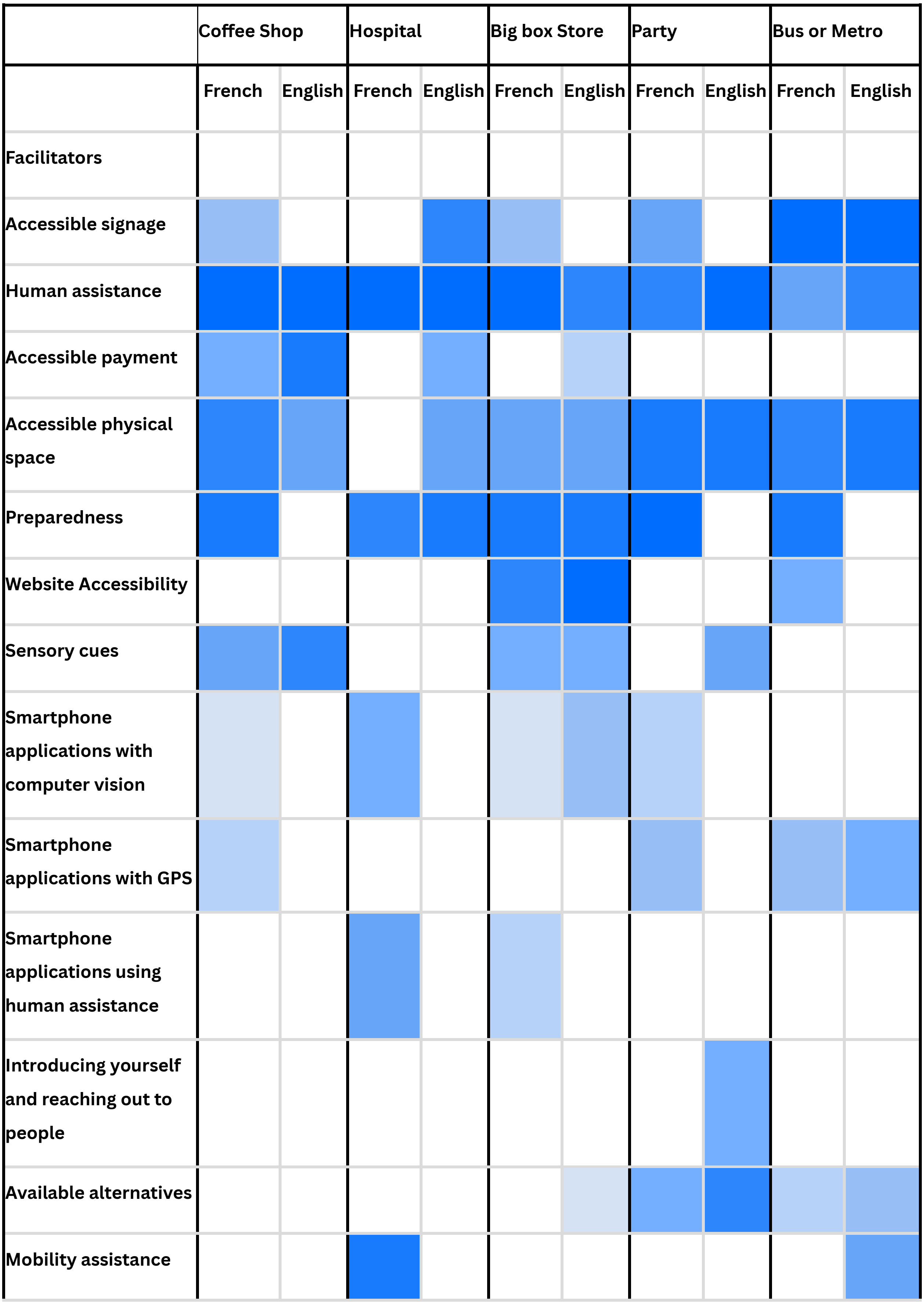
Coffee Shop
Hospital
Big box Store
Party
Bus or Metro
French
English
French
English
French
English
French
English
French
English
Facilitators
Accessible signage
Human assistance
Accessible payment
Accessible physical space
Preparedness
Website Accessibility
Sensory cues
Smartphone applications with computer vision
Smartphone applications with GPS
Smartphone applications using human assistance
Introducing yourself and reaching out to people
Available alternatives
Mobility assistance

	Coffee Shop		Hospital		Big box Store		Party		Bus or Metro	
	French	English	French	English	French	English	French	English	French	English
Obstacles										
Inaccessible signage										
Unsuccessful interactions										
Inaccessible payment										
Difficulties walking around										
Difficulty finding an exact location										
Inaccessible online information										
Multiplicity of items to be handled (Hands occupied)										
Difficulty finding a specific item										
Technology-related issues										
Difficulty finding a price or discount										
Difficulty finding human assistance										
Unexpected events										

**Noisy environment**										
**Difficulty finding what fits**										
**Other**		Lighting								

Rank	**1st**	**2nd**	**3rd**	**4th**	**5th**	**6th**	**7th**	**8th**	**9th**	**Unranked**
