# Supplementary material for: Perspectives From Canadian People With Visual Impairments in Everyday Environments Outside the Home: Qualitative Insights for Assistive Technology Development
Source: JMIR Rehabil Assist Technol. 2025 Jul 29;12:e73380. doi: 10.2196/73380 (PMC12306908; doi:10.2196/73380)
Supplement: Multimedia Appendix 5 [file rehab-v12-e73380-s005.docx]

|  | Coffee shop | | | | | |
| --- | --- | --- | --- | --- | --- | --- |
|  | Facilitators | | | | | |
| Rank | English Group | Average | | | French Group | Average |
|  |  |  | | |  |  |
| 1 | Human Assistance | 2.6 | | | Human Assistance | 1.9 |
| 2 | Preparedness | 3.4 | | | Accessible payment | 2.4 |
| 3 | Accessible physical space | 4.3 | | | Sensory cues | 2.6 |
| 4 | Sensory cues | 4.4 | | | Accessible physical space | 3.0 |
| 5 | Accessible payment | 4.7 | | |  |  |
| 6 | Accessible signage | 4.8 | | |  |  |
| 7 | Smartphone applications with GPS | 5.8 | | |  |  |
| 8 | Smartphone applications with computer vision | 6.0 | | |  |  |
|  |  |  | | |  |  |
|  | **Barriers** | | | | | |
|  |  |  | | |  |  |
| 1 | Difficulty finding an exact location | 2.7 | | | Lighting | 2.0 |
| 2 | Unsuccessful interactions | 3.1 | | | Inaccessible signage | 2.5 |
| 3 | Inaccessible signage | 3.8 | | | Difficulties walking around | 2.9 |
| 4 | Difficulties walking around | 4.4 | | | Inaccessible payment | 3.0 |
| 5 | Inaccessible payment | 4.8 | | | Difficulty finding an exact location | 3.0 |
| 6 | Noisy environment | 5.1 | | | Difficulty finding human assistance. | 4.0 |
| 7 | Hands occupied | 5.9 | | |  |  |
| 8 | Inaccessible online information | 6.2 | | |  |  |
|  |  | | | | | |
|  | **Hospital** | | | | | |
|  | Facilitators | | | | | |
| Rank | English Group | | Average | | French Group | Average |
|  |  | |  | |  |  |
| 1 | Human Assistance | | 1.4 | | Human Assistance | 2.1 |
| 2 | Mobility assistance | | 2.0 | | Preparedness | 2.1 |
| 3 | Preparedness | | 2.2 | | Accessible signage | 2.7 |
| 4 | Smartphone applications using human assistance | | 3.0 | | Accessible physical space | 3.3 |
| 5 | Smartphone applications with computer vision | | 3.7 | | Accessible payment | 4.8 |
|  |  | |  | |  |  |
|  | **Barriers** | | | | | |
|  |  | |  | |  | Average |
| 1 | Difficulty finding an exact location | | 1.9 | | Inaccessible signage | 2.4 |
| 2 | Unsuccessful interactions | | 2.3 | | Unsuccessful interactions | 2.9 |
| 3 | Difficulties walking around | | 2.6 | | Difficulties walking around | 3.1 |
| 4 | Inaccessible signage | | 3.2 | | Difficulty finding an exact location | 3.1 |
| 5 |  | |  | | Noisy environment | 4.8 |
| 6 |  | |  | | Multiplicity of items to be managed | 5.4 |
| 7 |  | |  | | Inaccessible payment | 6.4 |
|  |  | |  | |  |  |
|  | **Big box store** | | | | | |
|  | Facilitators | | | | | |
| Rank | English Group | Average | | | French Group | Average |
|  |  |  | | |  |  |
| 1 | Human assistance | 2.6 | | | Website accessibility | 1.9 |
| 2 | Preparedness | 3.9 | | | Preparedness | 2.5 |
| 3 | Website accessibility | 4.1 | | | Human assistance | 3.2 |
| 4 | Accessible physical space | 4.2 | | | Accessible physical space | 4.5 |
| 5 | Sensory cues | 4.3 | | | Sensory cues | 5.3 |
| 6 | Accessible signage | 5.1 | | | Smartphone Applications | 5.5 |
| 7 | Smartphone applications using human assistance | 5.6 | | | Accessible payment | 6.1 |
| 8 | Smartphone applications with computer vision | 6.2 | | | Alternatives available | 7.2 |
|  |  |  | | |  |  |
|  | **Barriers** | | | | | |
|  |  |  | | |  | Average |
| 1 | Difficulties walking around | 2.8 | | | Employee Assistance Request | 1.0 |
| 2 | Difficulty finding an exact location | 3.1 | | | Inaccessible signage | 2.4 |
| 3 | Difficulty finding a specific item | 3.6 | | | Difficulties walking around | 2.5 |
| 4 | Inaccessible signage | 4.0 | | | Unsuccessful interactions | 4.5 |
| 5 | Inaccessible payment | 4.0 | | | Multiplicity of items to manage | 4.5 |
| 6 | Difficulty finding a price or discount | 5.1 | | | Difficulty finding an exact location | 4.8 |
| 7 | Hands occupied | 5.4 | | | Inaccessible payment | 5.1 |
| 8 |  |  | | | Unforeseen events | 5.7 |
| 9 |  |  | | | Difficulty finding what fits | 7.4 |
|  |  |  | | |  |  |
|  | **Party** | | | | | |
|  | Facilitators | | | | | |
| Rank | English Group | Average | | French Group | | Average |
|  |  |  | |  | |  |
| 1 | Preparedness | 2.0 | | Human Assistance | | 1.2 |
| 2 | Accessible physical space | 2.4 | | Accessible physical space | | 2.7 |
| 3 | Human Assistance | 2.6 | | Alternatives available | | 3.1 |
| 4 | Accessible signage | 4.2 | | Sound sensory cues | | 3.6 |
| 5 | Alternatives available | 5.3 | | Introduce yourself and reach out to people | | 4.5 |
| 6 | Smartphone applications with GPS | 5.4 | |  | |  |
| 7 | Smartphone applications with computer vision | 6.0 | |  | |  |
|  |  |  | |  | |  |
|  | **Barriers** | | | | | |
|  |  |  | |  | | Average |
| 1 | Difficulties walking around | 2.6 | | Fear of spilling food or drink on others | | 2.0 |
| 2 | Unsuccessful interactions | 3.0 | | Noisy environment | | 2.4 |
| 3 | Noisy environment | 3.4 | | Difficulties walking around | | 2.5 |
| 4 | Multiple items to manage, while having no place to sit | 3.6 | | Unsuccessful interactions | | 2.6 |
| 5 | Difficulty finding an exact location | 4.1 | | Difficulty finding human assistance. | | 2.9 |
| 6 | Inaccessible signage | 4.3 | |  | |  |
|  |  |  | |  | |  |
|  | **Bus travel** | | | | | |
|  | Facilitators | | | | | |
| Rank | English Group | Average | | | French Group | Average |
|  |  |  | | |  |  |
| 1 | Accessible signage | 2.8 | | | Accessible signage | 1.9 |
| 2 | Preparedness | 3.0 | | | Accessible physical space | 2.4 |
| 3 | Accessible physical space | 3.0 | | | Human assistance | 2.8 |
| 4 | Human assistance | 3.2 | | | Mobility aid to get on the bus | 4.3 |
| 5 | Website accessibility | 5.1 | | | Smartphone applications with GPS | 4.6 |
| 6 | Smartphone applications with GPS | 5.1 | | | Alternatives available | 5.0 |
| 7 | Alternatives available | 5.8 | | |  |  |
|  |  |  | | |  |  |
|  | **Barriers** | | | | | |
|  |  |  | | |  | Average |
| 1 | Inaccessible signage | 2.1 | | | Inaccessible signage | 1.0 |
| 2 | Unsuccessful interactions with staff/patrons | 3.7 | | | Difficulty finding an exact location | 2.4 |
| 3 | Difficulties walking around | 3.9 | | | Unexpected events | 2.5 |
| 4 | Difficulty finding a specific item | 4.2 | | | Difficulty finding a specific item | 4.5 |
| 5 | Unexpected events | 4.6 | | | Technical problems | 4.5 |
| 6 | Inaccessible online information | 4.7 | | |  |  |
| 7 | Inaccessible payment | 4.9 | | |  |  |
