# Supplementary material for: Perspectives From Canadian People With Visual Impairments in Everyday Environments Outside the Home: Qualitative Insights for Assistive Technology Development
Source: JMIR Rehabil Assist Technol. 2025 Jul 29;12:e73380. doi: 10.2196/73380 (PMC12306908; doi:10.2196/73380)
Supplement: Multimedia Appendix 6 [file rehab-v12-e73380-s006.docx]

| Theme | | Participant | Scenario | Quote |
| --- | --- | --- | --- | --- |
| **Facilitator** | | | | |
|  | |  |  |  |
| Human Assistance | | AF37 | Coffee Shop | “I already know the kind of the prices on what they sell, and so I just ask them, what do you have today?” |
| Preparation | | AF29  AF37 | Coffee Shop | “If I do, prior research, just to make sure that I just map out the trajectory. So, I know where the established in the establishment”  “Sometimes what I will do is try to read the menu online beforehand”. |
| Accessibility of physical space | | AF28 | Coffee Shop | “If the tables and chairs are fixed it does not moves giving no surprises due to their movement and they are placed widely” |
| Sensory cues | | AF37  AF03 | Coffee Shop | “For me sometimes, like in a go into a coffee shop is the smell of the coffee that can queue me to where the door is, and sometimes also like the hearing the door squeak and the one I have gone”  “I'm a bit lucky at the there is a bell at the main entrance door, so each time someone gets in the bell like you sound the bell” |
| Accessible payment | | AF37 | Coffee Shop | “Usually my phone works, and I can see the payment right away on my screen reader” |
| Accessible signage | | AF29 | Coffee Shop | “I think the names of the surrounding businesses like while using an app like soundscape. If you can have the names of the surrounding businesses before adjacent. After that it usually helps to locate the business.”  “If there is a signage that clearly says it, just order here.” |
| Smartphone applications | | AF03 | Coffee Shop | “I have downloaded some apps made up. Not all of them like McDonald’s, Tim Horton’s. It's almost in my computer and my cell phone. So, I can browse it, and then make the orders through it, and also help as well with the cash you. It helps a lot. Sometimes I get the promotion and everything.” |
|  | |  |  |  |
| **Barriers** | | | | |
|  | |  |  |  |
| Precise location | | A10  AF08 | Coffee Shop | “The precise location, like the street, address, and the suite number. and for going into the establishment to know. But there are steps, how many steps, whether there is a stair, whether there is an elevator.”  “Locating where the terminal is. It's never in a standard place that is close to the customer. Sometimes you must reach and try to find where it is.” |
| Difficulty finding human assistance | | A10 | Coffee Shop | “People get very upset If you get into the line at the wrong spot.”  “It's very difficult to get employees to turn around from what they are from. Well, they, the employees, are up at account to face in public. It's very difficult to get them to turn around and start reading a menu on the wall.” |
| Inaccessible signage | | A07  A10 | Coffee Shop | “The sign is up too high. People can't read it from when they're coming up the street, or the print on the sign can be very small very hard to see, even with magnification.”  “The clarity of the signage is also very important. Sometimes the signs are created using a certain format of writing. They're not really printed. They use the more artistic format I think that can be. Make it difficult to read.” |
| Difficulties walking around | | AF29 | Coffee Shop | “Finding a table with vision loss and with a mobility as sometimes the corridors are narrow, and the tables are just kind of all bunched up together.” |
| Inaccessible payment | | AF37 | Coffee Shop | “I have to enter the card, and sometimes not very often they ask for a PIN, and sometimes the machine is inaccessible. It has a touch keypad that I cannot use myself, and so I would have to even trust them to put in the PIN for me, which is something I really wouldn't want to do.” |
| High ambient noise | | AF08  AF29 | Coffee Shop | “Sometimes the environment is so loud when you're asking a question.”  “Sometimes environments can be very noisy. So, it is quite difficult to hear, and this is quite difficult to be heard.” |
| Hands occupied | | AF37 | Coffee Shop | “Well for me it makes it harder that I have to hold my cane in one hand, and then it may be like if I have a coffee and a sandwich, or something. In the other hand, it's kind of impossible, and then locate the chair. It's a different nightmare. So, what I usually do is ask one of the employees to point me at a correct table, or to even help me carry whatever I need to carry to the table where I need to sit, because, holding the cane on the food and trying to feel around with no vision. It is not easy.” |
| Lighting | | A07 | Coffee Shop | “Lighting could be poor around the entrance to the establishment. So, you may miss the doorway going in, especially if it's into the evening hours.” |
| Difficult in entry and exit | | AF03 | Coffee Shop | “Sometimes to get to the entrance. You have to go through the parking lot, which is basically difficult for us to go around. We've people coming and going. Sometimes the door is just right on the parking.” |
|  | |  |  |  |
| **Facilitator** | | | | |
|  | |  |  |  |
| Helpful staff | | A07  AF28 | Hospital | “Sometimes it's best just to go to security on the ground floor and say, `I’m legally blind. I need to find some help.”  “Ask the Security Guard to go to the right floor. Then ask the receptionist and tell him or her. When the doctor comes to call my name, she needs to come to me instead of just showing up the number.” |
| Preparation | | A07 | Hospital | “One thing to do would be to go the day before to visit the place, and then you can avoid all the problems with navigation by getting familiar with the place.” |
| Smartphone applications using human assistance | | A10 | Hospital | “There are certain documents online, you can point it out to the registration desk.” |
| Smartphone applications with computer vision | | A10 | Hospital | “You can use an app to help you read the room signs.” |
|  | |  |  |  |
| **Barriers** | | | | |
|  | |  |  |  |
| Problems finding a precise location | | A07 | Hospital | “You’ve got to find the right floor. Just find the elevator. There are all kinds of all kinds of challenges with that, if you want to go a big place like, say, St. Michael's Hospital, and you got to go up to floor 8 or 10.” |
| Unsuccessful interactions with staff | | AF29 | Hospital | “Unfortunately, the receptionist is there. Many of them do not get up to show me where to go. They just say it's easy to find this number and I have no clue.” |
| Inaccessible payment | | A07 | Hospital | “While paying with debit or with credit card you need to press the keys and those with poor vision will have difficulty pressing the keys.” |
| Inaccessible online information | | AF37 | Hospital | “Sometimes you might have to fill out a form, which could be easier if they had the form online which is why I need to ask for some help.” |
| Inaccessible physical space | | A07 | Hospital | “Finding the reception desk is tricky, it's very rarely in the front.” |
|  | |  |  |  |
| **Facilitator** | | | | |
|  | |  |  |  |
| Helpful staff/patrons | | AF29  A10 | Big Box Store | “An agency that helps me with staff. The people who were paid to do such things with me, especially the shopping cart. That's a huge use.”  “If you're making comparison between items and prices. I find that having human intervention really helps.” |
| Preparation | | AF29 | Big Box Store | “Preparing in advance. That's a facilitator, just and so for some stores I can eat entire. The website will tell me on what aisle, and how many of the items are left?” |
| Website accessibility | | A10 | Big Box Store | “If you do the online shopping it can be easier to find the items online, just put it into a cartoon line and schedule the delivery, and then customer service will cancel. They do the free charge for you and the items. The groceries come the following day.” |
| Accessibility of physical space | | AF26 | Big Box Store | “Each department in the department store would have a different surface, as far as the let's say the aisles, so that if I want to go to the sports article sections. They would tell me to follow the carpet so I would with my cane follow the carpet. If it's to kitchen utensils, they would say, let's say, follow floor type tiles, so that would be for me about the only way I would venture on my own in such a place.” |
| Accessibility of signage | | A10 | Big Box Store | “If the numbers are very large and in large print. They may be high up for what that helps as well.” |
| Smartphone applications using human assistance | | AF37 | Big Box Store | “I would prepare look online, if possible, do my shopping online if I can. If not, I would either get someone to assist me directly at the counter, and I would ask them to read me the information on the on the different products, or I also use something like be my eyes, which is a paid a subscription app for assistance, visual assistance.” |
|  | |  |  |  |
| **Barriers** | | | | |
|  | |  |  |  |
| Problems locating a precise item | | AF33 | Big Box Store | “One thing that makes it difficult for me is, is it's either the top row or completely the bottom row.” |
| Inaccessible signage | | AF33 | Big Box Store | “Some of the departments are not labeled. There's no sign above from the far in which you can tell. Which. Where is it? So, you have to go and walk around to be able to figure out where it is.” |
| Inaccessible checkout | | AF33 | Big Box Store | “I can't use self-service because the machines don't talk the way they should. and I would need to have help anyway.” |
| Problems identifying a price or sale | | AF33 | Big Box Store | “Using your phone to see the price tag. It's not practical because the phone is often sometimes too far to be able to read it.” |
| Hands occupied | | AF37 | Big Box Store | “I have to hold my can in one hand. And so, if I had to take a basket. maybe, or just carry my own backpack so I carry carts mostly.” |
|  | |  |  |  |
| **Facilitator** | | | | |
|  | |  |  |  |
| Helpful friends | | AF29  AF37 | Party with Friends | “Just asking the person, where is your house in the apartment? And can you just turn the light on, or just maybe I need you downstairs or something.”  “I usually call when I’m arriving, and how my friend, or whoever can you please meet me downstairs? That'd be pretty helpful, because I usually won't know where the house is.” |
| Smartphone Apps | | AF37 |  | “I use my phone to call my friend or use one of those apps already mentioned to try to figure out where the exact entrance is.” |
|  | |  |  |  |
| **Barriers** | | | | |
|  | |  |  |  |
| Difficulties walking around | | AF29 | Party with Friends | “If the party is on the fourth floor of a building anymore. We have all these like spiral staircases. It is impossible to see the address.” |
| Unsuccessful interactions with friends | | AF37 | Party with Friends | “For me is the challenge is that in some kind, of course, people are moving around all the time, you know, going from group to group to group to group, and it's very over overwhelming to kind of follow that it's someone I I know, and even work with there's people that I don't know, because I cannot pinpoint, or it's very hard for me to kind of just join a random group.” |
| High ambient noise | | AF08 | Party with Friends | “The biggest obstacle is a very loud party, so the music is very, very loud, and then you cannot identify people based on their voice. You cannot.” |
| Multiple items to manage, while having no place to sit | | AF08 | Party with Friends | “For me it's not necessarily the number of people attending the party, but enough seats for the number of people invited.” |
|  | |  |  |  |
| **Facilitator** | | | | |
|  | |  |  |  |
| Accessibility of signage | | A07 | Bus/ Metro | “Well, in Toronto we have a TTC type of service where it announces 52. Lawrence was to Pearson Airport, or whatever. So, when the bus pulls up, it announces what number that is, and where that bus is going.” |
| Preparation | | A10 | Bus/ Metro | “There's a lot of research to be done ahead of time. You have to know the stop numbers. You have to know the intersections where those bus stops are. These are things you have to know before getting there.” |
| Accessibility of physical space | | AF03  AF33 | Bus/ Metro | “You can fill up your card in pharmacies and all the convenient stores, you know, convenience stores in Montreal.”  “Sitting on the side of the door near the driver would help.” |
| Helpful staff/patrons | | AF37 | Bus/ Metro | “I usually do either ask if someone else is waiting it in the back of the backstop to make sure that they tell me that that's the right bus or go into the bus and ask the driver to confirm that I’m taking the right bus.” |
| Smartphone applications with GPS | | AF37 | Bus/ Metro | “I also use my app like any of the transit apps to show. That shows you when the box does so. I kind of can't know. Okay, this is this bus coming. It's supposed to be my bus. But sometimes you're not on time, and it's one right after you so I’m always trying to confirm with the driver or the other people around.” |
|  | |  |  |  |
| **Barriers** | | | | |
|  | |  |  |  |
| Inaccessible signage | | AF33 | Bus/ Metro | “One thing is that the sign is way too high. It's sometime 8 to 12, and even 14 feet high. I can't reach it by phone. Be able to see if I’m at the right spot.” |
| Unsuccessful interactions with staff/patrons | | AF26 | Bus/ Metro | “Besides the metro stations because I am looking for a teller that will fill in my card. And even at the metro station they do not do it anymore. You have to go to the machine. Every station has a different location for the machine. It's a pain.” |
| Difficulties walking around | | AF33 | Bus/ Metro | “We enter from the front and exit from the back, and sometimes the bus was crowded, making it difficult to walk through the back door, and we have to push something to get out to get down the stairs, and then, with the door opening.” |
| Problems locating a precise item | | AF29 | Bus/ Metro | “Where's the button depending on the seat. It can be up. It can be down. It can be somewhere. It can be on the pole so it's just locating the ghost, darn thing is quite a feat.” |
| Unexpected events | | AF37 | Bus/ Metro | “They do have that audio announcement, but sometimes it might happen that it's out of sync.” |
| Inaccessible online information | | AF29 | Bus/ Metro | “There is not the website of the bus company. After 10 min conversation with someone at customer service, they said they've been struggling for 10 years. Even if they have some website, it's not always updated.” |
| Inaccessible payment | | AF29 | Bus/ Metro | “…if I’m paying with coins. Yes, this is an issue because I don't know where to dump the coins.” |
|  | |  |  |  |
| Theme (Use of smartphone) | | | | |
| Participant | Quote | | | |
|  |  | | | |
| F23 | “There are a lot of applications. It would be fun in an ideal world to have an app that will get the best of all the other apps. At some point, you don't know which one to open, it becomes heavy. It would be fun to have something that looks for the good sides (of the apps that exist). It would help us in our daily lives” | | | |
| AF26 | “Training is the bigger obstacle for me, the biggest one. Even if there's group training sessions. You can't have a one-on-one appointment with your rehab center, because it takes a long time, and you can't just ask your neighbor, because he doesn't know that. So Blind square for me is very useful to know where I am, but I still don't know how to put in my address to figure out how to get there” | | | |
| AF37 | “If the app would allow me to do the thing without needing human assistance. I would rather have the app, because, of course, sometimes it is good to interact with people, and maybe it might be quicker, and someone can help you and all that sometimes. You might not feel like talking to anyone, or you just want to do your thing without having to interrupt any anyone doing something, or you just don't feel like” | | | |
| AF29 | “Everything with voice is making my tasks less of a pain” | | | |
| AF33 | “Switching from one tab to another, one function to another, it's not user friendly” | | | |
| AF33 | “Most apps don't have proper accessibility levels like, let's say, a back button. Sometimes it doesn't say back button. It says a program or string thing which doesn't mean anything for someone who doesn't see or doesn't see well enough. it irritates me greatly” | | | |
| AF08 | “I think we are living in it at a time where there are so many options, so many apps, and each of them is incomplete. So, you have to use a multitude of apps. You know the scanning one to get sort of. You know what the sign says. You have the navigation, the turn by turn. Many of them are complicated like I find blind square, very complicated. So, there isn't one app that gives you everything” | | | |
| AF08 | “But also, I want to talk about, working hands-free when you are on the street I have it problem having to pull out my phone and click on this and click on that. So, I want to raise the issue of affordability nowadays, you know, as a companion to an app. You have, like those smart glasses like an envision glasses which is very expensive” | | | |
